# Clinical decision-making competencies and work readiness in senior nursing students: a path analysis study

**DOI:** 10.1186/s12909-026-08614-z

**Published:** 2026-02-12

**Authors:** Sümeyye Akçoban, Soner Berşe, Serap Özdemi̇r, Betül Tosun

**Affiliations:** 1 Health Services Department, Kırıkhan Vocational School, Hatay Mustafa Kemal University, Alparslan Türkeş Street, 31440 Kırıkhan , Türkiye Hatay, Turkey; 2 Department of Nursing, Faculty of Health Sciences, Gaziantep University, Gaziantep, Türkiye Turkey; 3 Faculty of Nursing, Hacettepe University, Türkiye Sümeyye AKÇOBAN, Adnan Saygun Street, D-Block 1st Floor, RN, Lecturer Ankara, Türkiye

**Keywords:** Clinical Decision-Making, Work readiness, Senior nursing students, Professional competence

## Abstract

**Background:**

Clinical decision-making and work readiness are essential competencies that support nursing students’ transition into professional practice. Understanding the factors that are associated with these competencies is critical for designing effective educational strategies and planning future internship programs.

**Purpose:**

This study aimed to assess the clinical decision-making competencies and work readiness levels of senior nursing students and to examine the factors associated with these competencies using path analysis.

**Materials and methods:**

The study included 219 senior nursing students enrolled in a nursing program in Turkey. Data were collected using a Sociodemographic Data Form, the Clinical Decision-Making in Nursing Scale (CDMNS), and the Work Readiness Scale (WRS). Statistical analyses included descriptive statistics, t-tests, ANOVA, and path analysis modeling performed using SPSS and AMOS.

**Results:**

The mean age was 22.4 ± 2.3 years and 74% were female. The majority (78.5%) had completed two terms of internship. Students reported moderate clinical decision-making competence (CDMNS mean = 3.34 ± 0.43) and moderate-to-high work readiness (WRS mean = 6.10 ± 1.44). Female students and those who had voluntarily chosen nursing reported higher CDMNS scores. Consistent use of scientific evidence, confidence in patient-safety decisions, tolerance of ambiguity, and generating alternative solutions were associated with higher scores. In the path model, Organizational Intelligence showed the strongest positive associations with all CDMNS subdimensions (β = 0.37–0.67), while Personal Work Characteristics showed negative associations (β = −0.14 to − 0.35).

**Conclusion:**

Senior nursing students exhibited moderate levels of clinical decision-making competence and work readiness. Personal motivation, evidence-based practice, creative problem-solving, and organizational skills were associated with clinical decision-making competence and work readiness. Strengthening structured internship programs and promoting innovative thinking may further enhance students’ readiness for professional practice.

**Clinical trial number:**

Not applicable.

## Contributions to the Literature

- This study is among the few investigations in Turkey that comprehensively examine the relationship between clinical decision-making competence and work readiness among senior nursing students and evaluate this relationship using path modeling.
- The findings demonstrate that scientific evidence use, decision-making in ambiguity, confidence in patient safety decisions, and creative problem-solving significantly associated with both clinical decision-making and work readiness relationships rarely examined in the existing literature.
- The study provides practical insights for nursing educators and policymakers by highlighting the need to strengthen internship programs and evidence-based practice training, supporting students’ smoother transition into professional nursing roles.

## Introduction

Clinical decision-making is a cognitive and analytical process that enables nurses to analyze information related to patient care and make safe, effective, and evidence-based interventions [1]. Clinical decision-making consists of assessing the patient, interpreting data, selecting the appropriate intervention, and monitoring outcomes. This skill is of critical importance for nursing practice, patient safety, and quality of care [2, 3].

Nursing students in their final year of nursing education, who participate directly in patient care during the transition to the profession, must have well-developed clinical decision-making skills [4]. These skills support final-year nursing students’ ability to apply theoretical knowledge, adapt to the clinical environment, and make safe and independent decisions [5]. It has been reported that nursing students’ clinical decision-making skills are mostly at the ‘analysis’ level, meaning that they make decisions based on a specific logical sequence and information [6].

In final-year nursing students, advanced clinical decision-making competence is closely related to the level of “work readiness” they should possess when starting their career [7]. Preparation for work refers to the student feeling cognitively, emotionally, and behaviorally ready for the professional responsibilities they will encounter during the clinical practice process; that is, it refers to being competent in fundamental areas such as professional knowledge, skills, self-confidence, problem solving, and communication [8]. This level of preparation facilitates student nurses’ adaptation during the transition to the profession and contributes to more effective and safer practices in patient care [9]. Additionally, it enhances students’ resilience and performance in busy and stressful clinical environments, positively impacting professional identity development and safe care behaviors [10]. Students with a high level of work readiness are seen to have more advanced clinical decision-making, communication, and self-management skills and to be more successful in adapting to their professional roles [11]. Recent studies have further indicated that individual characteristics, particularly emotional intelligence, play a significant role in the development of clinical decision-making competence. Evidence suggests that higher levels of emotional intelligence are associated with more effective clinical decision-making among nurses and nursing students in diverse clinical settings [12–14]. From a conceptual perspective, clinical decision-making and work readiness are interrelated competencies that jointly shape nursing students’ transition from education to professional practice [1, 8]. While clinical decision-making reflects students’ cognitive and analytical ability to provide safe and evidence-based care [1, 3], work readiness encompasses their perceived preparedness to apply these skills effectively within complex clinical environments [8, 9]. Insufficient integration of these competencies may compromise patient safety, professional confidence, and early career adaptation, particularly during the internship period when students assume increased responsibility in patient care [10, 11].

A review of the literature reveals that some studies have separately addressed the clinical decision-making competencies and work readiness levels of student nurses [2, 4, 5, 12]. Eskiyurt and Özkan (2024) found that student nurses’ clinical decision-making levels were above average and reported that collaborative learning had a positive effect on clinical decision-making competence [4]. İlaslan et al. (2023), on the other hand, found that critical thinking and clinical decision-making skills had developed to a significant degree among nursing students, with students’ performance in these areas being above average [2]. Factors affecting the clinical decision-making level of final-year nursing students were examined, and it was found that students’ decision-making competencies were generally above average and that factors such as self-confidence and mentoring support were decisive [5]. Chen et al. (2024) found that nursing students’ level of readiness for work was generally high and reported that factors such as age, voluntary choice of nursing as a subject, student leadership experience, and confidence in clinical practice significantly influenced readiness for work [15]. In addition, recent studies have examined the relationship between emotional intelligence and clinical decision-making across different nursing populations, including nursing students, neonatal intensive care nurses, and critical care nurses. These studies consistently report positive associations between emotional intelligence and clinical decision-making competence, underscoring the multifactorial nature of clinical reasoning in nursing practice [12–14]. Although previous studies have examined clinical decision-making competence and work readiness among nursing students separately, the interaction between these two critical competencies during the transition to professional practice remains insufficiently explored. In particular, there is a lack of evidence focusing on senior nursing students who function as intern nurses and actively participate in patient care. Therefore, this study aimed to examine the clinical decision-making competence and work readiness levels of senior nursing students and to identify factors associated with these competencies.

### The hypotheses of the study

H1: Senior nursing students’ clinical decision-making competence is significantly associated with their level of work readiness.

## Materials and methods

### Study design

This analytical cross-sectional study employed path analysis to examine the associations between clinical decision-making competencies and work readiness. The study was conducted with senior nursing students (enrolled in the 8th semester) at a state university in the Southeast Anatolia region of Turkey between October 16 and November 1, 2025. The STROBE checklist was used to report the research [16].

### Study population and sample

The study population consisted of all final-year nursing students enrolled in the 8th semester at a state nursing department (*n* = 250). Nursing internships are conducted in the 8th semester, and students’ clinical decision-making competencies and work readiness levels were assessed during this period, prior to their transition to the profession. No sampling method was used in this study; the aim was to reach the entire population. This approach reduces the risk of selection bias, prevents sampling errors, and allows the results to be representative of the population as a whole. Thus, the internal validity of the study was strengthened, and the generalizability of the findings was increased. Including all students in the study allowed for a more comprehensive presentation of existing differences in terms of individual, educational, and clinical factors. This allowed for obtaining more reliable and comprehensive data on the clinical decision-making competencies and work readiness levels of the intern nurses [17, 18].

The sample size of 219 participants was considered adequate for path analysis with four explanatory variables and four dependent variables, following recommendations of 10–20 observations per estimated parameter. Effect sizes were calculated using Cohen’s f² (f² = R²/[1 − R²]) and interpreted as small (0.02), medium (0.15), or large (0.35). The model explained substantial variance in the outcome variables, with effect sizes ranging from medium to large: Evaluating Consequences (R² = 0.365, f² = 0.575, large), Exploring Options (R² = 0.314, f² = 0.458, large), Questioning Goals (R² = 0.256, f² = 0.344, medium), and Information Seeking (R² = 0.185, f² = 0.227, medium). These effect sizes indicate practically meaningful associations between work readiness dimensions and clinical decision-making competence.

### Inclusion criteria

The study included nursing students who voluntarily chose to participate, were actively enrolled in the 8th semester of their program, and completed all items of the survey, ensuring a comprehensive representation of their experiences and competencies.

### Data collection tools

For data collection, the “Student Demographic Information Form,” the “Clinical Decision-Making in Nursing Scale,” and the “Work Readiness Scale” were used.

### Student demographic information form

A form developed by the researchers based on the literature was used to collect information on students’ age, gender, voluntary choice of nursing profession, and 15 statements related to their clinical decision-making and work readiness [4, 5, 10].

### Clinical Decision-Making in Nursing Scale (CDMNS)

This scale, developed by Jenkins (1983) and adapted into Turkish by Durmaz-Edeer and Sarıkaya (2015), aims to describe the behavior of nursing students in patient care situations [19, 20]. The scale consists of 40 items and four subdimensions rated on a 5-point Likert scale (1 = never, 5 = always). The subdimensions of the scale are Exploring Options and Ideas, Questioning Goals and Values, Evaluating Consequences, and Information Seeking and Unbiased Assimilation. Eighteen items (2, 4, 6, 12, 13, 15, 19, 21, 22, 23, 24, 25, 30, 31, 32, 34, 39, 40) are reverse scored. Subdimension scores range from 10 to 50, and total scores range from 40 to 200. The scale has no cutoff point; higher scores indicate higher decision-making competence, and lower scores indicate lower competence. The Turkish version of the CDMNS had a Cronbach’s α of 0.78. In this study, the total internal consistency of the CDMNS was Cronbach’s α = 0.89. The Cronbach’s alpha values for the subdimensions were: Exploring Options and Ideas, α = 0.80; Questioning Goals and Values, α = 0.78; Evaluating Consequences, α = 0.73; and Information Seeking and Unbiased Assimilation, α = 0.71. The Clinical Decision-Making in Nursing Scale (CDMNS) was selected because it is a well-designed instrument specifically designed to assess clinical decision-making behaviors among nursing students in patient care situations. The scale has been widely used in nursing education research and has demonstrated acceptable psychometric properties in different cultural contexts. As the Turkish validity and reliability of the scale had been previously established and its internal consistency reported in earlier studies, a pilot study was not required in the present research.

### Work Readiness Scale (WRS)

The WRS, developed by Caballero et al. (2011) and adapted for nurses by Walker et al. (2013) with validated reliability, was used to assess nurses’ work readiness [21]. The scale consists of 46 items across four subdimensions: Work Competence, Social Intelligence, Organizational Intelligence, and Personal Work Characteristics, rated from 0 (Strongly Disagree) to 10 (Strongly Agree). Beyhan and Ergün conducted the Turkish adaptation, with Cronbach’s alpha values ranging from 0.85 to 0.93 [8]. Higher scores in Work Competence, Social Intelligence, and Organizational Intelligence and lower scores in Personal Work Characteristics indicate higher work readiness. In this study, the total internal consistency of the WRS was Cronbach’s α = 0.97, with subdimension values as follows: Work Competence, α = 0.95; Social Intelligence, α = 0.93; Organizational Intelligence, α = 0.96; and Personal Work Characteristics, α = 0.89. The Work Readiness Scale (WRS) was chosen due to its comprehensive structure that evaluates work readiness across multiple dimensions relevant to professional nursing practice, including competence, social and organizational intelligence, and personal work characteristics. The scale has been previously validated for use in nursing populations and adapted into Turkish with strong reliability evidence. Given that the Turkish adaptation and psychometric validation of the scale had already been completed and reliability coefficients were reported in previous studies, a pilot study was not required for the present research.

### Data collection

In this study, data were collected via an online survey created by the researchers using the Google Forms application. Before the data collection process, the student representatives of the final-year nursing department were contacted and informed in detail about the purpose of the study, that participation was entirely voluntary, that the information collected would be kept confidential, and that it would be used solely for scientific purposes. The survey link was then shared via the class representatives on the social media group. At the beginning of the survey, the purpose of the research, data confidentiality within the framework of ethical rules, the principle of voluntariness, and how the information would be used were presented to the participants clearly and understandably. The researchers’ contact details were added to the form in case participants had any questions about the process. It took participants approximately 10–15 min to complete the survey. No personal identification information was requested during the survey, and all responses were stored in a secure digital environment accessible only to the research team members. Control questions were integrated into the survey questions to assess the accuracy and attention levels of participants’ responses.

### Conceptual framework

This study was guided by a conceptual framework developed based on existing literature and theoretical assumptions regarding the relationship between work readiness and clinical decision-making competence among senior nursing students. In this framework, work readiness is conceptualized as a multidimensional construct encompassing Work Competence, Social Intelligence, Organizational Intelligence, and Personal Work Characteristics. These dimensions are assumed to be associated with clinical decision-making competence, which is operationalized through the subdimensions of the Clinical Decision-Making in Nursing Scale (CDMNS), including Exploring Options and Ideas, Questioning Goals and Values, Information Seeking and Unbiased Assimilation, and Evaluating Consequences.

Sociodemographic and educational characteristics (e.g., gender, voluntary choice of profession, use of scientific evidence, self-confidence in decision-making, shift work experience, and perceived adequacy of education) are positioned as contextual variables that may be associated with both work readiness and clinical decision-making competence. Based on this framework, it was hypothesized that higher levels of work readiness dimensions would be associated with higher levels of clinical decision-making competence. This conceptual structure informed the specification of the path analysis model tested in the present study.

### Ethical considerations

Before commencing the research, ethical approval was obtained from the Non-Interventional Research Ethics Committee of Hatay Mustafa Kemal University (date: 08.10.2025; meeting number: 13; Decision No no. 30). Additionally, the necessary institutional permission was obtained from the faculty of health sciences where the research will be conducted (Number: E-50581566-020-734430). The data collection form was prepared online and included information at the beginning about the purpose of the study, that students could leave the study at any time, that the information shared would be kept confidential, and that the data obtained would not be used for purposes other than the study itself. Students’ voluntary participation in the study and their consent were obtained through the option ‘I voluntarily agree to participate in the study’ in the first item of the questionnaire. This study was conducted in accordance with the principles of the Declaration of Helsinki. Necessary permissions from the authors of the scales to be used were obtained via email before the research began.

### Data analysis

The data were analyzed using SPSS version 26.0. Before analysis, data integrity, missing values, and outliers were examined, and the normality of continuous variables was assessed using skewness and kurtosis coefficients. Descriptive statistics were summarized using frequencies, percentages, means, and standard deviations. Preliminary analyses were conducted to examine associations between clinical decision-making competence, work readiness, and selected sociodemographic and educational variables. These analyses informed the specification of the path model.

### Path analysis

Path analysis was conducted to examine the associations between work readiness dimensions and clinical decision-making competence in accordance with the proposed conceptual framework. In this analysis, the subscale scores of the Work Readiness Scale (Work Competence, Social Intelligence, Organizational Intelligence, and Personal Work Characteristics) and the subdimensions of the Clinical Decision-Making in Nursing Scale (Exploring Options and Ideas, Questioning Goals and Values, Information Seeking and Unbiased Assimilation, and Evaluating Consequences) were treated as observed variables.

The model was estimated using maximum likelihood estimation. Sociodemographic and educational characteristics (age, gender, grade point average, family income status, internship experience, and shift/on-call work experience) were examined in preliminary analyses but were not retained as covariates in the final path model for parsimony, as the primary aim was to examine direct associations between work readiness and clinical decision-making dimensions. Model fit was evaluated using the chi-square test (χ²), Comparative Fit Index (CFI), Goodness-of-Fit Index (GFI), and Root Mean Square Error of Approximation (RMSEA). Standardized path coefficients (β), confidence intervals, and explained variance (R²) values were reported.

## Results

The mean age of participants was 22.42 ± 2.33 years (range: 21–46). The majority were female (74%), reported a medium family income (84.5%), and had voluntarily chosen the nursing profession (65.3%). More than half had experience with rotating shifts (58.4%), and approximately half reported confidence in clinical decision-making and patient safety–related decisions (Table 1).

**Table 1:** Descriptive characteristics of the study sample (*n* = 219)

Variable	Category	*n*	%
Age (years)	Mean ± SD = 22.42 ± 2.33 (min–max: 21–46)		
Gender	Male	57	26.0
	Female	162	74.0
Family income status	Low	24	11.0
	Medium	185	84.5
	High	10	4.6
Shift / night-shift working experience	No, it never happened.	63	28.8
	Yes, only the day shift	28	12.8
	Yes, I worked shifts, including night shifts.	128	58.4
Voluntary Choice of Nursing Profession	No	76	34.7
	Yes	143	65.3
Use of scientific evidence in clinical decisions	No	7	3.2
	Partial	90	41.1
	Yes	122	55.7
Self-confidence in decisions affecting patient safety	No	10	4.6
	Partial	94	42.9
	Yes	115	52.5
Ability to decide in ambiguous clinical situations	No	8	3.7
	Partial	102	46.6
	Yes	109	49.8
Developing alternative solutions during practice	No	6	2.7
	Partial	91	41.6
	Yes	122	55.7
Feeling ready to start nursing after graduation	No	20	9.1
	Partial	114	52.1
	Yes	85	38.8
Belief in ability to perform necessary nursing interventions in emergencies	No	16	7.3
	Partial	123	56.2
	Yes	80	36.5
Belief in ability to adapt to teamwork	No	5	2.3
	Partial	51	23.3
	Yes	163	74.4
Perceived adequacy of professional knowledge and skills for starting work	No	21	9.6
	Partial	117	53.4
	Yes	81	37.0
Effect of nursing education on safe clinical decision-making	Low	17	7.8
	Medium	140	63.9
	High	62	28.3
Effect of nursing education on readiness to start the profession	No	21	9.6
	Partial	122	55.7
	Yes	76	34.7

Senior nursing students demonstrated moderate-to-high levels across all CDMNS subscales, with mean scores ranging from 3.27 to 3.43, indicating consistent clinical decision-making competence. The overall CDMNS mean score was 3.34 (SD = 0.43). Work readiness levels were also high across all WRS subscales, with mean scores ranging from 5.29 (Personal Work Characteristics) to 6.58 (Organizational Intelligence), and an overall mean score of 6.10 (SD = 1.44). All CDMNS and WRS subscales demonstrated good-to-excellent internal consistency (Cronbach’s α = 0.710–0.965) (Table 2).

**Table 2:** Mean scores and internal consistency of the clinical Decision-Making and work readiness scales (*n* = 219)

Scale / Subscale	No. of Items	Range	Mean	SD	Cronbach’s α
Clinical Decision-Making in Nursing Scale (CDMNS)					
Exploring options and ideas	10	1–5	3.43	0.60	0.800
Questioning goals and values	10	1–5	3.27	0.53	0.788
Evaluating consequences	10	1–5	3.40	0.55	0.732
Information seeking and unbiased assimilation	10	1–5	3.27	0.50	0.710
CDMNS Total Score	40	1–5	3.34	0.43	0.897
Work Readiness Scale (WRS)					
Work competence	14	0–10	5.90	1.71	0.958
Social intelligence	8	0–10	6.30	1.82	0.934
Organizational intelligence	16	0–10	6.58	1.77	0.965
Personal work characteristics¹	8	0–10	5.29	1.73	0.891
WRS Total Score	46	0–10	6.10	1.44	0.971

Table 3 presents the distribution of clinical decision-making competencies and work readiness scores across sociodemographic and professional characteristics. Gender differences were primarily evident at the CDMNS subscale level. Female students scored significantly higher than males in the Exploring Options (t = − 2.32, *p* = .022), Questioning Goals and Values (t = − 2.24, p = .026), and Evaluating Consequences (t = − 2.20, *p* = .029) subscales. Consistent with these findings, the total CDMNS score was also significantly higher among female students compared to males (3.39 ± 0.42 vs. 3.21 ± 0.43, t = − 2.77, *p* = .006). In contrast, no significant gender differences were observed across WRS subscales (p > .05). Voluntary choice of the nursing profession was associated with higher scores in selected dimensions. Students who voluntarily chose nursing had higher CDMNS scores in Exploring Options (*p* = .039) and Questioning Goals and Values (p = .025), whereas Information Seeking did not differ significantly (*p* = .179). For work readiness, voluntary choice was associated with higher WRS total score (p = .011) and Work Competence (*p* = .005); differences in Social Intelligence and Organizational Intelligence were marginal (*p* = .057 and *p* = .053, respectively), and Personal Work Characteristics did not differ significantly (*p* = .187). Use of scientific evidence in clinical decision-making showed a consistent graded association across both instruments. Students who consistently used scientific evidence reported higher scores on the CDMNS (F = 12.19, p < .001) and across all WRS subscales (F = 17.37–21.84, all *p* < .001), with the strongest association observed in the Organizational Intelligence subscale (Yes: 7.01 ± 1.70; Partial: 6.24 ± 1.48; No: 3.49 ± 2.69; F = 18.47, *p* < .001) (Table 3).

**Table 3:** Comparison of clinical Decision-Making and work readiness scores by sociodemographic characteristics (*n* = 219)

Characteristics	CDMNS Total M ± SD	Exploring Options M ± SD	Questioning Goals M ± SD	Evaluating Consequences M ± SD	Information Seeking M ± SD	WRS Total M ± SD	Work Competence M ± SD	Social Intelligence M ± SD	Organizational Intelligence M ± SD	Personal Work M ± SD
Gender										
Female	3.39 ± 0.42	3.48 ± 0.61	3.32 ± 0.50	3.45 ± 0.56	3.31 ± 0.49	6.09 ± 1.41	5.85 ± 1.70	6.28 ± 1.81	6.59 ± 1.76	5.31 ± 1.68
Male	3.21 ± 0.43	3.27 ± 0.57	3.14 ± 0.58	3.26 ± 0.49	3.16 ± 0.52	6.14 ± 1.53	6.05 ± 1.77	6.37 ± 1.86	6.55 ± 1.82	5.25 ± 1.89
*t* / *p*	***t = -2.77***, p = .006**	***t = -2.32***, p = .022*	***t = -2.24***, p = .026*	***t = -2.20***, p = .029*	*t = -1.95*,* p = .052*	*t = 0.23*,* p = .817*	*t = 0.75*,* p = .453*	*t = 0.30*,* p = .768*	*t = -0.14*,* p = .888*	*t = -0.22*,* p = .829*
**Voluntary Choice of Nursing Profession**										
No	3.27 ± 0.47	3.31 ± 0.65	3.16 ± 0.58	3.41 ± 0.56	3.21 ± 0.45	5.76 ± 1.47	5.46 ± 1.65	5.98 ± 1.87	6.26 ± 1.83	5.08 ± 1.70
Yes	3.38 ± 0.41	3.49 ± 0.57	3.33 ± 0.49	3.40 ± 0.54	3.30 ± 0.52	6.28 ± 1.40	6.13 ± 1.71	6.48 ± 1.78	6.75 ± 1.72	5.41 ± 1.75
*t* / *p*	*t = -1.74*,* p = .083*	***t = -2.08***, p = .039*	*t = -2.26*, p = .025*	*t = 0.19*,* p = .850*	*t = -1.35*,* p = .179*	*t = -2.56*, p = .011*	*t = -2.83*, p = .005**	*t = -1.91*,* p = .057*	*t = -1.94*,* p = .053*	*t = -1.32*,* p = .187*
**Family Income Status**										
Lower	3.30 ± 0.38	3.35 ± 0.42	3.21 ± 0.60	3.39 ± 0.54	3.24 ± 0.53	5.44 ± 1.86	4.87 ± 1.96	5.46 ± 2.23	6.02 ± 2.15	5.24 ± 1.91
Equal	3.35 ± 0.42	3.44 ± 0.60	3.29 ± 0.50	3.39 ± 0.54	3.27 ± 0.47	6.19 ± 1.28	6.03 ± 1.55	6.40 ± 1.65	6.65 ± 1.65	5.34 ± 1.63
Higher	3.36 ± 0.78	3.35 ± 0.96	3.12 ± 0.82	3.64 ± 0.67	3.35 ± 0.94	6.05 ± 2.51	5.93 ± 3.04	6.59 ± 3.12	6.60 ± 2.78	4.64 ± 2.94
*F* / *p*	*F = 0.14*,* p = .870*	*F = 0.29*,* p = .746*	*F = 0.67*,* p = .511*	*F = 1.00*,* p = .369*	*F = 0.16*,* p = .848*	*F = 2.96*,* p = .054*	***F = 5.04***, p = .007**	*F = 2.98*,* p = .053*	F = 1.35, *p* = .261	*F = 0.78*,* p = .460*
**Shift/Rotating Work Schedule**										
No, never worked	3.52 ± 0.39	3.69 ± 0.49	3.40 ± 0.37	3.66 ± 0.59	3.34 ± 0.39	6.41 ± 1.11	6.15 ± 1.47	6.76 ± 1.65	7.22 ± 1.43	4.88 ± 2.00
Yes, only daytime	3.31 ± 0.48	3.40 ± 0.63	3.23 ± 0.72	3.27 ± 0.52	3.31 ± 0.59	6.31 ± 1.55	6.27 ± 1.84	6.65 ± 1.79	6.42 ± 1.74	5.85 ± 1.67
Yes, rotating including nights	3.26 ± 0.42	3.30 ± 0.61	3.22 ± 0.54	3.30 ± 0.49	3.23 ± 0.53	5.90 ± 1.53	5.70 ± 1.78	6.00 ± 1.86	6.30 ± 1.86	5.37 ± 1.57
*F* / *p*	***F = 8.28***, p < .001**	***F = 9.23***, p < .001**	*F = 2.76*,* p = .065*	***F = 10.44***, p < .001**	*F = 1.30*,* p = .276*	***F = 3.03***,*** p = .050***	*F = 2.25*,* p = .107*	***F = 4.33***, p = .014*	**F = 6.15**, p = .003**	***F = 3.41***, p = .035*
**Use of Scientific Evidence in Clinical Decisions**										
No	2.80 ± 0.57	2.71 ± 0.90	2.40 ± 0.97	3.20 ± 0.31	2.90 ± 0.79	3.40 ± 2.36	3.05 ± 2.25	3.68 ± 2.75	3.49 ± 2.69	3.54 ± 2.00
Partial	3.25 ± 0.36	3.27 ± 0.53	3.20 ± 0.35	3.30 ± 0.53	3.21 ± 0.43	5.81 ± 1.25	5.49 ± 1.50	5.82 ± 1.59	6.24 ± 1.48	5.49 ± 1.66
Yes	3.44 ± 0.44	3.58 ± 0.58	3.38 ± 0.56	3.48 ± 0.56	3.34 ± 0.52	6.47 ± 1.30	6.37 ± 1.60	6.81 ± 1.71	7.01 ± 1.70	5.25 ± 1.72
*F* / *p*	***F = 12.19***, p < .001**	*F = 13.19*, p < .001**	***F = 14.37***, p < .001**	***F = 3.33***, p = .038*	*F = 3.71*, p = .026*	*F = 21.84*, p < .001**	*F = 19.72*, p < .001**	*F = 17.37*, p < .001**	*F = 18.47*, p < .001**	*F = 4.36*, p = .014*
**Self-Confidence in Patient Safety Decisions**										
No	3.14 ± 0.68	3.27 ± 0.90	2.71 ± 0.93	3.51 ± 0.59	3.07 ± 0.73	4.40 ± 2.40	4.09 ± 2.50	4.50 ± 2.77	4.84 ± 2.68	3.96 ± 1.82
Partial	3.27 ± 0.38	3.30 ± 0.56	3.24 ± 0.42	3.33 ± 0.51	3.23 ± 0.46	5.69 ± 1.17	5.33 ± 1.28	5.74 ± 1.46	6.08 ± 1.49	5.49 ± 1.31
Yes	3.42 ± 0.44	3.54 ± 0.59	3.35 ± 0.53	3.45 ± 0.57	3.32 ± 0.51	6.59 ± 1.32	6.53 ± 1.69	6.92 ± 1.75	7.14 ± 1.68	5.25 ± 1.98
*F* / *p*	***F = 4.03***, p = .019*	*F = 4.54*, p = .012*	*F = 7.66*, p < .001**	*F = 1.54*, *p = .218*	***F = 1.63***, ***p = .198***	***F = 20.62***, p < .001**	***F = 22.14***, p < .001**	***F = 18.73***, p < .001**	***F = 16.49***, p < .001**	***F = 3.68***, p = .027*
**Ability to Decide in Ambiguous Clinical Situations**										
No	2.97 ± 0.65	3.06 ± 0.89	2.65 ± 1.02	3.36 ± 0.48	2.80 ± 0.65	4.06 ± 2.55	3.76 ± 2.67	4.00 ± 2.68	4.48 ± 2.88	3.83 ± 2.10
Partial	3.25 ± 0.33	3.30 ± 0.54	3.21 ± 0.37	3.27 ± 0.44	3.23 ± 0.44	5.68 ± 1.24	5.33 ± 1.37	5.86 ± 1.60	6.05 ± 1.52	5.40 ± 1.31
Yes	3.45 ± 0.47	3.57 ± 0.61	3.38 ± 0.57	3.52 ± 0.62	3.35 ± 0.52	6.64 ± 1.24	6.59 ± 1.61	6.89 ± 1.71	7.23 ± 1.62	5.30 ± 2.00
*F* / *p*	***F = 9.58***, p < .001**	***F = 6.86***, p = .001**	***F = 9.46***, p < .001**	***F = 5.64***, p = .004**	***F = 5.42***, p = .005**	***F = 24.33***, p < .001**	***F = 25.42***, p < .001**	***F = 17.35***, p < .001**	***F = 20.83***, p < .001**	***F = 3.11***, p = .047*
**Effect of Nursing Education on Readiness to Start Profession**										
No	3.22 ± 0.56	3.29 ± 0.80	3.01 ± 0.78	3.39 ± 0.62	3.18 ± 0.63	4.82 ± 1.98	4.51 ± 2.04	4.84 ± 2.00	5.14 ± 2.29	4.72 ± 1.74
Partial	3.31 ± 0.39	3.39 ± 0.55	3.25 ± 0.42	3.37 ± 0.52	3.24 ± 0.48	5.93 ± 1.15	5.61 ± 1.33	6.04 ± 1.52	6.45 ± 1.51	5.34 ± 1.53
Yes	3.43 ± 0.44	3.53 ± 0.62	3.38 ± 0.58	3.45 ± 0.57	3.35 ± 0.49	6.73 ± 1.38	6.75 ± 1.78	7.14 ± 1.85	7.19 ± 1.75	5.38 ± 2.02
*F* / *p*	*F = 2.67*, * p = .072*	*F = 1.85*, *p = .160*	*F = 4.35*, p = .014*	*F = 0.52*, *p = .593*	*F = 1.54*, *p = .216*	***F = 19.21***, p < .001**	***F = 21.33***, p < .001**	***F = 18.64***, p < .001**	***F = 13.04***, p < .001**	***F = 1.28***, ***p = .279***
**Developing Alternative Solutions During Practice**										
No	3.06 ± 0.86	3.15 ± 1.16	2.52 ± 1.18	3.67 ± 0.60	2.90 ± 0.83	3.85 ± 2.87	3.63 ± 2.96	3.29 ± 2.90	4.21 ± 3.27	4.08 ± 3.01
Partial	3.23 ± 0.32	3.28 ± 0.45	3.17 ± 0.36	3.27 ± 0.47	3.21 ± 0.45	5.66 ± 1.20	5.31 ± 1.38	5.84 ± 1.61	6.04 ± 1.48	5.30 ± 1.49
Yes	3.44 ± 0.45	3.55 ± 0.65	3.39 ± 0.55	3.48 ± 0.58	3.33 ± 0.51	6.54 ± 1.31	6.45 ± 1.64	6.80 ± 1.69	7.10 ± 1.68	5.35 ± 1.82
*F* / *p*	***F = 7.68***, p < .001**	*F = 5.98*, p = .003**	***F = 12.16***, p < .001**	***F = 4.61***, p = .011*	***F = 3.40***, p = .035*	***F = 20.66***, p < .001**	***F = 19.80***, p < .001**	***F = 17.99***, p < .001**	***F = 17.03***, p < .001**	***F = 1.53***, ***p = .219***
Post-hoc (Tukey)	Yes > Partial**	NS	Yes > Partial*	All differ**	Yes > Partial**	Yes > Partial, No**	Yes > Partial, No**	Yes > Partial, No**	Yes > Partial, No**	Yes > Partial, No**
**Feeling Ready to Start Nursing After Graduation**										
No	3.20 ± 0.56	3.17 ± 0.76	3.02 ± 0.77	3.41 ± 0.56	3.18 ± 0.65	4.98 ± 1.92	4.39 ± 1.93	4.73 ± 2.12	5.54 ± 2.36	5.16 ± 2.06
Partial	3.32 ± 0.40	3.37 ± 0.56	3.28 ± 0.45	3.37 ± 0.56	3.24 ± 0.46	5.90 ± 1.22	5.57 ± 1.34	6.08 ± 1.51	6.41 ± 1.61	5.29 ± 1.52
Yes	3.41 ± 0.43	3.56 ± 0.59	3.32 ± 0.54	3.44 ± 0.53	3.33 ± 0.51	6.63 ± 1.37	6.69 ± 1.74	6.97 ± 1.85	7.06 ± 1.70	5.32 ± 1.93
*F* / *p*	*F = 2.53*, *p = .082*	*F = 4.51*, p = .012*	*F = 2.73*, *p = .068*	*F = 0.33*, *p = .719*	*F = 1.21*, *p = .301*	***F = 14.50***, p < .001**	***F = 22.77***, p < .001**	***F = 15.91***, p < .001**	***F = 7.54***, p < .001**	***F = 0.07***,***p = .933***
Post-hoc (Tukey)	NS	NS	NS	NS	NS	Yes > Partial, No**	Yes > Partial, No**	Yes > Partial*	Yes > Partial, No**	Yes > Partial, No**
**Belief in Ability to Perform Nursing Interventions in Emergencies**										
No	3.30 ± 0.41	3.43 ± 0.47	3.00 ± 0.62	3.51 ± 0.53	3.26 ± 0.44	5.40 ± 1.62	4.88 ± 1.64	5.29 ± 2.03	5.83 ± 2.02	5.55 ± 1.77
Partial	3.30 ± 0.38	3.34 ± 0.54	3.29 ± 0.44	3.35 ± 0.51	3.21 ± 0.46	5.83 ± 1.20	5.53 ± 1.40	6.01 ± 1.55	6.35 ± 1.59	5.10 ± 1.46
Yes	3.42 ± 0.50	3.55 ± 0.70	3.30 ± 0.62	3.46 ± 0.60	3.37 ± 0.56	6.67 ± 1.56	6.67 ± 1.88	6.96 ± 1.97	7.08 ± 1.88	5.54 ± 2.07
*F* / *p*	*F = 2.06*, *p = .130*	***F = 2.85***, ***p = .060***	*F = 2.36*, *p = .097*	*F = 1.40*, *p = .248*	*F = 2.56*, * p = .080*	***F = 11.31***, p < .001**	***F = 15.73***, p < .001**	***F = 9.92***, p < .001**	***F = 5.80***, p = .004**	*F = 1.77*, * p = .172*
Post-hoc (Tukey)	NS	Yes > Partial*	NS	NS	NS	Yes > Partial, No**	Yes > Partial, No**	Yes > Partial*	Yes > Partial, No**	NS
**Belief in Ability to Adapt to Teamwork**										
No	2.72 ± 0.70	2.68 ± 0.97	2.30 ± 1.20	3.32 ± 0.36	2.58 ± 0.69	3.26 ± 2.83	2.86 ± 2.59	3.20 ± 3.06	3.11 ± 2.84	4.33 ± 3.24
Partial	3.08 ± 0.21	3.06 ± 0.39	3.06 ± 0.26	3.11 ± 0.33	3.10 ± 0.42	5.62 ± 1.26	5.28 ± 1.45	5.68 ± 1.53	5.97 ± 1.43	5.43 ± 1.47
Yes	3.44 ± 0.43	3.56 ± 0.58	3.37 ± 0.52	3.49 ± 0.58	3.34 ± 0.49	6.34 ± 1.31	6.19 ± 1.63	6.59 ± 1.73	6.88 ± 1.68	5.28 ± 1.76
*F* / *p*	***F = 22.66***, p < .001**	***F = 20.87***, p < .001**	***F = 17.75***, p < .001**	***F = 10.49***, p < .001**	***F = 10.32***, p < .001**	***F = 17.08***, p < .001**	***F = 15.31***, p < .001**	***F = 13.70***, p < .001**	***F = 17.00***, p < .001**	*F = 0.94*, *p = .392*
Post-hoc (Tukey)	Yes > Partial, No**	Yes > Partial, No**	Yes > Partial, No**	Yes > Partial, No**	Yes > Partial, No*	Yes > Partial, No*	Yes > Partial, No*	Yes > Partial, No*	Yes > Partial, No*	NS
**Perceived Adequacy of Professional Knowledge and Skills**										
No	3.31 ± 0.61	3.34 ± 0.81	3.05 ± 0.76	3.58 ± 0.68	3.26 ± 0.68	4.90 ± 1.78	4.51 ± 1.75	4.79 ± 2.01	5.32 ± 2.20	4.82 ± 1.93
Partial	3.29 ± 0.36	3.35 ± 0.53	3.26 ± 0.44	3.36 ± 0.48	3.21 ± 0.43	5.90 ± 1.20	5.56 ± 1.39	6.07 ± 1.58	6.49 ± 1.55	5.12 ± 1.48
Yes	3.42 ± 0.46	3.56 ± 0.63	3.35 ± 0.57	3.41 ± 0.59	3.36 ± 0.53	6.71 ± 1.39	6.75 ± 1.75	7.04 ± 1.79	7.03 ± 1.80	5.67 ± 1.96
*F* / *p*	*F = 2.22*, * p = .111*	***F = 3.45***, p = .034*	*F = 2.97*, *p = .054*	*F = 1.51*, *p = .222*	*F = 2.02*, *p = .135*	***F = 18.39***, p < .001**	***F = 22.85***, p < .001**	***F = 17.16***, p < .001**	***F = 8.64***, p < .001**	*F = 3.40*, p = .035*
Post-hoc (Tukey)	NS	Yes > Partial*	NS	NS	NS	Yes > Partial, No**	Yes > Partial, No**	Yes > Partial, No*	Yes > Partial, No**	NS
**Effect of Nursing Education on Safe Clinical Decision-Making**										
Low	3.13 ± 0.55	3.12 ± 0.75	2.85 ± 0.81	3.36 ± 0.60	3.18 ± 0.70	4.67 ± 2.08	4.38 ± 2.33	4.68 ± 2.33	5.00 ± 2.37	4.48 ± 2.14
Moderate	3.30 ± 0.37	3.38 ± 0.53	3.25 ± 0.38	3.36 ± 0.51	3.21 ± 0.44	5.97 ± 1.22	5.72 ± 1.41	6.12 ± 1.59	6.46 ± 1.58	5.26 ± 1.51
High	3.49 ± 0.48	3.61 ± 0.67	3.44 ± 0.65	3.50 ± 0.61	3.42 ± 0.54	6.80 ± 1.32	6.73 ± 1.78	7.17 ± 1.78	7.28 ± 1.68	5.60 ± 2.01
*F* / *p*	***F = 6.75***, p = .001**	***F = 5.56***, p = .004**	***F = 9.34***, p < .001**	*F = 1.33*, *p = .266*	***F = 4.16***, p = .017*	***F = 19.15***, p < .001**	***F = 16.87***, p < .001**	***F = 16.43***, p < .001**	***F = 13.26***, p < .001**	*F = 2.92*, *p = .056*
Post-hoc (Tukey)	High > Low*	High > Low*	High > Moderate*	NS	High > Low*	High > Moderate, Low**	High > Moderate, Low**	High > Moderate, Low**	High > Moderate, Low**	NS

*CDMNS*  Clinical Decision-Making in Nursing Scale, *WRS*  Work Readiness Scale, *M*  Mean, *SD*  Standard Deviation, *NS*  Not Significant. *p < .05; **p < .01. Post-hoc comparisons conducted using Tukey HSD test where applicable

Self-confidence in patient safety decisions was significantly associated with work readiness, with strong associations observed across all WRS dimensions (F = 16.49–22.14, all *p *< .001). This variable was also associated with clinical decision-making competence in the CDMNS total score (F = 4.03, *p* = .019), Exploring Options (F = 4.54, *p* = .012), and Questioning Goals and Values (F = 7.66, *p* = .001) subscales. Students who reported being able to make decisions in ambiguous clinical situations exhibited significantly higher scores across multiple CDMNS subscales (F = 5.42–9.58, *p* < .01) as well as across all WRS dimensions (F = 17.35–25.42, all *p* < .001). Shift work experience was associated with differences in clinical decision-making competence; students without shift work experience reported higher CDMNS total scores than those with rotating shifts (3.52 ± 0.39 vs. 3.26 ± 0.42; F = 8.28, *p* < .001). Shift work was also associated with WRS dimensions, including Social Intelligence (F = 4.33, *p* = .014), Organizational Intelligence (F = 6.15, *p* = .003), and Personal Work Characteristics (F = 3.41, *p* = .035). The perceived effect of nursing education on readiness demonstrated strong associations with work readiness; students who perceived high educational effect reported significantly higher scores across all WRS dimensions (F = 11.35–19.15, all *p* < .001). In addition, the ability to develop alternative solutions during clinical practice was significantly associated with both overall clinical decision-making competence (CDMNS total: F = 7.68, *p* = .001) and all work readiness subscales (F = 17.03–20.66, all *p* < .001) (Table 3).

Path analysis revealed differential associations between work readiness dimensions and clinical decision-making subdimensions. Organizational Intelligence showed the strongest and most consistent positive associations with all four CDMNS subdimensions: Evaluating Consequences (β = 0.673, *p* < .001), Exploring Options and Ideas (β = 0.513, *p* < .001), Information Seeking (β = 0.422, *p* < .001), and Questioning Goals and Values (β = 0.372, *p* < .001). Personal Work Characteristics showed significant negative associations with all CDMNS subdimensions: Evaluating Consequences (β = −0.352, *p* < .001), Exploring Options and Ideas (β = −0.244, *p* < .001), Information Seeking (β = −0.200, *p* = .002), and Questioning Goals and Values (β = −0.140, *p* = .020). Work Competence demonstrated a mixed pattern, with a significant positive association with Questioning Goals and Values (β = 0.249, *p* = .027) but a significant negative association with Evaluating Consequences (β = −0.229, *p* = .028). Social Intelligence showed no significant associations with any CDMNS subdimension (all *p* > .05). The model explained 36.5% of the variance in Evaluating Consequences (R² = 0.365), 31.4% in Exploring Options and Ideas (R² = 0.314), 25.6% in Questioning Goals and Values (R² = 0.256), and 18.5% in Information Seeking (R² = 0.185). Global fit indices for the path model are presented in Table 4 and 5.

**Table 4:** Path model fit indices (*n* = 219)

Index	Value	Recommended Criterion
χ²	0.00	non-significant preferred
df	4	—
χ²/df	0.00	< 3.0
CFI	1.000*	≥ 0.95
GFI	0.999	≥ 0.95
RMSEA	< 0.001	< 0.06
SRMR	< 0.001	< 0.08

*CFI reached its upper bound (1.00) because model discrepancy was near zero (χ² ≈ 0) in this low-degrees-of-freedom path model (df = 4); incremental fit indices should be interpreted with caution in such models

**Table 5:** Path analysis results: standardized associations between work readiness dimensions (WRS) and clinical Decision-Making subdimensions (CDMNS) (*n* = 219)

CDMNS	WRS	β	*p*	*R*²
Exploring Options and Ideas	Work Competence	−0.068	0.528	0.314
	Social Intelligence	0.095	0.416	
	Organizational Intelligence	0.513***	< 0.001	
	Personal Work Characteristics	−0.244***	< 0.001	
Questioning Goals and Values	Work Competence	0.249*	0.027	0.256
	Social Intelligence	−0.094	0.441	
	Organizational Intelligence	0.372***	< 0.001	
	Personal Work Characteristics	−0.140*	0.020	
Evaluating Consequences	Work Competence	−0.229*	0.028	0.365
	Social Intelligence	0.027	0.807	
	Organizational Intelligence	0.673***	< 0.001	
	Personal Work Characteristics	−0.352***	< 0.001	
Information Seeking	Work Competence	0.116	0.321	0.185
	Social Intelligence	−0.134	0.293	
	Organizational Intelligence	0.422***	< 0.001	
	Personal Work Characteristics	−0.200**	0.002	

*β  *standardized path coefficient*, R² *proportion of variance explained. *p < .05, **p < .01, ***p < .001

**Fig. 1:**
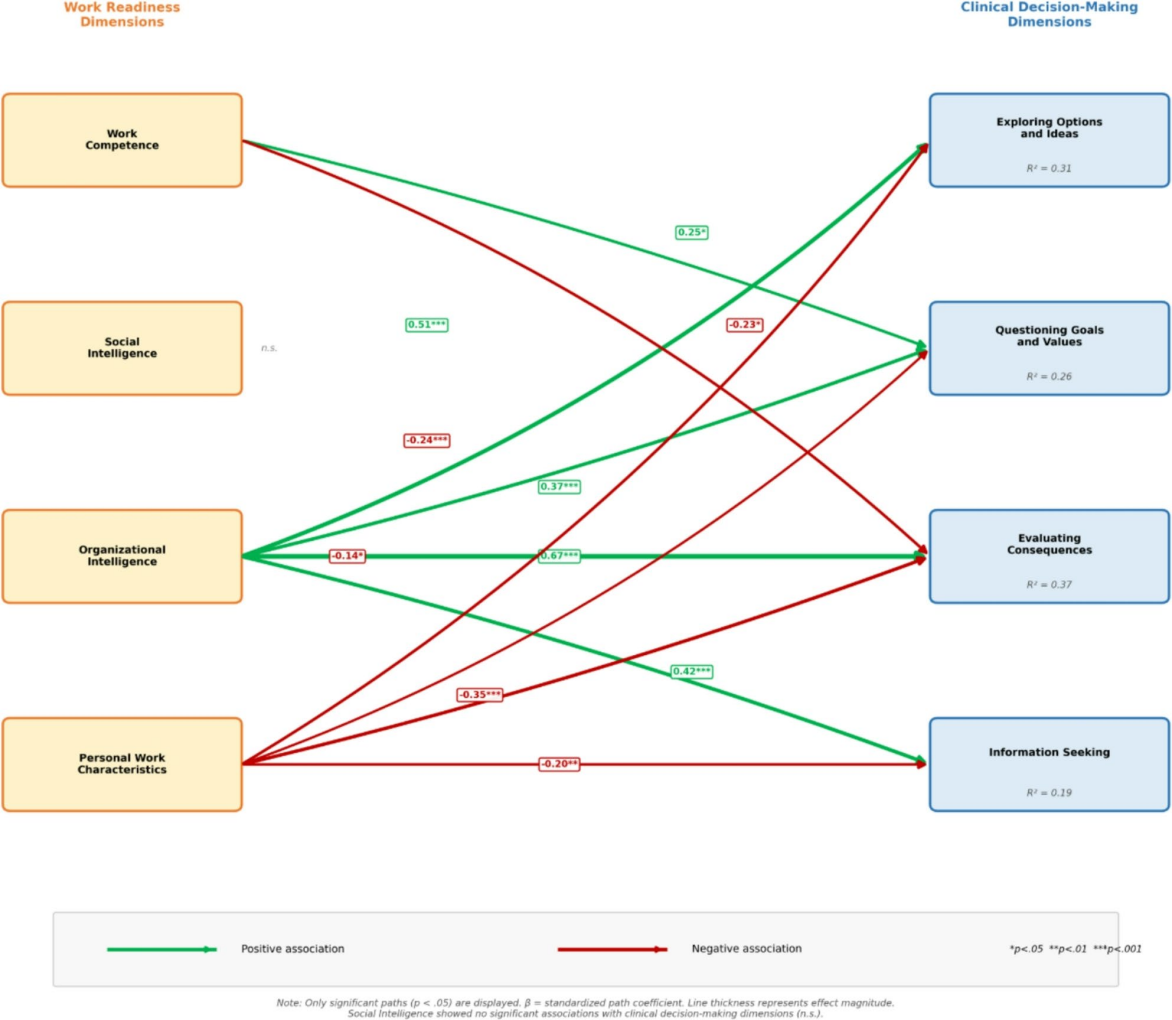
Path diagram showing standardized associations between work readiness dimensions and clinical decision-making subdimensions. Note: Only significant paths (*p* < .05) are displayed. Positive associations shown in green, negative associations in red. Line thickness indicates effect magnitude. β = standardized path coefficient. * < .05, **p < .01, ***p < .001

The path model showed near-perfect global fit indices (χ²(4) = 0.00, *p* > .999; χ²/df = 0.00; CFI = 1.000; GFI = 1.000; RMSEA < 0.001 [90% CI: 0.000–0.000]; SRMR < 0.001). However, given the low degrees of freedom (df = 4) resulting from the flexible model specification (16 direct paths with freed residual covariances), these indices should be interpreted with caution. In such low-df models, a near-perfect fit is expected and does not necessarily indicate model superiority; therefore, interpretation should focus primarily on the magnitude and significance of individual path coefficients and explained variance (R²) values rather than global fit indices. All 16 direct paths from work readiness subdimensions to clinical decision-making subdimensions were estimated, and residual covariances among selected outcome variables were freely estimated (Table 5). Organizational Intelligence showed the largest standardized coefficient in relation to the Evaluating Consequences subscale, and this association reached statistical significance (β = 0.673, *p* < .001). Overall, the model explained a meaningful proportion of variance across the CDMNS subscales, as presented in Table 5; Fig. 1.

## Discussion

This study examined the work readiness of senior nursing students prior to their transition into professional practice in relation to their clinical decision-making competence. The findings indicated that students demonstrated a moderate level of clinical decision-making competence, consistent with previous research suggesting that clinical reasoning develops progressively throughout nursing education [2, 4, 22].

Female students reported higher clinical decision-making competence scores than male students. This finding contrasts with Günerigök et al. (2020), who reported higher scores among males and associated this difference with perceived autonomy and confidence [6]. The present results may reflect changing gender dynamics in nursing education or sample-specific characteristics. These findings suggest that gender-related patterns in clinical decision-making competence may vary across educational and cultural contexts, highlighting the need for further research to understand underlying factors.

Students who voluntarily chose the nursing profession and those who reported using scientific evidence during clinical decision-making demonstrated higher competence scores. This finding aligns with studies indicating that evidence-based practice is associated with enhanced analytical reasoning and more systematic clinical judgment [6]. Professional motivation combined with evidence-based reasoning appears to be associated with greater engagement in complex decision-making processes.

Students who reported being able to make decisions in uncertain clinical situations also demonstrated higher clinical decision-making competence, supporting the view that tolerance of uncertainty is an important component of clinical reasoning [1]. In contrast, lower competence scores observed among students engaged in shift work represent an important contextual finding. Prior studies have shown that shift work, particularly night shifts, is associated with reduced attention and cognitive performance [6, 23]. Accordingly, work-related fatigue and circadian disruption may be associated with challenges in maintaining optimal decision-making competence during clinical education.

With respect to work readiness, senior nursing students demonstrated a moderate level of preparedness, consistent with findings from previous studies involving students approaching graduation [11, 15]. While this suggests acquisition of core professional competencies, it also indicates a potential need for additional support during the transition to professional practice. Students who voluntarily chose nursing and who reported using scientific evidence during clinical decisions demonstrated higher work readiness. These findings are consistent with Ersoy and Ayaz-Alkaya (2024), who highlighted the association between professional identity, intrinsic motivation, and readiness for practice [11]. Evidence-based decision-making may also be associated with higher self-efficacy, which in turn relates to greater perceived readiness for clinical responsibilities [6].

Students with higher confidence in their clinical decision-making also reported greater work readiness. Previous research has shown that confidence is associated with more effective decision implementation and adaptive performance in complex care environments [24, 25]. These findings suggest that self-confidence may facilitate students’ ability to manage uncertainty, contributing to a smoother transition into professional practice.

Path analysis indicated that work readiness dimensions were differentially associated with subdimensions of clinical decision-making competence. Notably, the Questioning Goals and Values subdimension highlighted the relevance of personal values and professional attitudes in clinical reasoning. This observation is consistent with evidence suggesting that self-efficacy and professional self-concept are associated with decision-making performance [26, 27]. Aboalrob et al. (2025) similarly reported that a strong professional self-concept is related to higher clinical decision-making competence [26].

Furthermore, the Organizational Intelligence dimension accounted for a substantial proportion of variance in the Evaluating Consequences sub-dimension of clinical decision-making. This finding highlights the importance of organizational structure and learning climate in influencing students’ evaluative judgments. Previous studies have reported associations between positive clinical learning environments and academic motivation, whereas inadequate environments have been linked to challenges in clinical reasoning and decision-making [28, 29]. Thus, the relatively high decision-making scores observed in this study may be related to supportive organizational learning conditions.

Contrary to expectations, the Social Intelligence dimension did not show statistically significant associations with any CDMNS subdimension in the path analysis (all *p* > .05), although bivariate correlations indicated moderate positive relationships (*r* = .28–0.43). This suggests that when controlling for other work readiness dimensions, social–cognitive skills may not directly contribute to clinical decision-making competence. Nevertheless, simulation-based education and interactive learning environments have been associated with improved decision-making confidence and self-efficacy through feedback and peer interaction [30, 31]. Moreover, educational interventions that enhance self-efficacy have been linked to improved clinical decision-making performance among nursing students [32].

### Limitations

This study had certain limitations. First, the sample was selected from a single institution, which may limit the generalizability of the findings to other nursing departments or educational settings. Second, as the data were collected through self-reporting, there is a possibility of response bias, particularly regarding perceived competence and readiness for work. Third, as some subgroups (e.g., students with full-placement experience) were small, the power to detect differences within these groups may be limited. Finally, factors such as institutional policies, mentoring quality, and differences in clinical settings were not examined; however, these elements may also influence clinical decision-making competence and job readiness.

### Conclusion and future directions

Senior nursing students demonstrated moderate levels of clinical decision-making competence and work readiness. These competencies were associated with personal motivation, voluntary choice of the nursing profession, use of scientific evidence, confidence in patient safety–related decisions, and creative problem-solving abilities. In addition, organizational intelligence and outcome evaluation were strongly associated with clinical decision-making competence, underscoring the importance of supportive educational and organizational contexts during the transition to professional practice.

Based on these findings, nursing curricula and internship programs may benefit from integrating structured evidence-based practice training, such as guided literature appraisal activities and case-based clinical reasoning exercises. Creative problem-solving skills could be strengthened through simulation-based scenarios that encourage alternative solution generation in complex and uncertain clinical situations. Furthermore, fostering organizational intelligence through mentorship programs, interprofessional teamwork experiences, and orientation activities that enhance understanding of institutional processes may support students’ evaluative judgment and professional adaptation.

Future research should employ longitudinal designs to examine how clinical decision-making competence and work readiness develop over time and to clarify the temporal nature of these associations. Multi-institutional studies across different regions are recommended to enhance generalizability. Additionally, intervention-based studies targeting evidence-based practice skills, creative problem-solving, and organizational intelligence during undergraduate nursing education may provide valuable insights into strategies that facilitate a smoother transition from education to professional nursing practice.

In this context, the following actions are proposed:

#### Structured internship experience

- Expanding and strengthening clinical placement programs to provide practical and hands-on learning.
- Ensuring that students gain experience in varied clinical settings, including high-acuity and emergency care.

#### Evidence-Based practice and problem solving

- Incorporating evidence-based practice training into the curriculum to enhance clinical decision-making.
- Promotion of creative problem-solving exercises and simulations to improve professional competence.

#### Organizational skills and work readiness

- Providing workshops and mentorship to strengthen organizational intelligence and outcome evaluation skills.
- Supporting students’ confidence and autonomy in making clinical decisions through guided practices.

Implementing these strategies may enhance students’ readiness for professional practice, facilitate a smoother transition from student to competent nurse, and be associated with improvements in the overall quality of care in clinical settings.

## Data Availability

The datasets generated and/or analyzed during the current study are available from the corresponding author on reasonable request.

## References

[1] Standing, M. Clinical judgement and decision making in nursing (3rd ed.). Sage Publications. 2017. 10.1111/j.1365-2648.2007.04583.x 18352971

[2] İlaslan E, Adıbelli D, Teskereci G, Üzen Cura Ş. Development of nursing students’ critical thinking and clinical decision-making skills. Teach Learn Nurs. 2023;18(1):152–9. 10.1016/J.TELN.2022.07.004.

[3] Saban M, & Dubovi I. A comparative vignette study: Evaluating the potential role of a generative AI model in enhancing clinical decision-making in nursing. Journal of Advanced Nursing. 2025;81(11):7489–99. 10.1111/jan.16101 10.1111/jan.16101 PMC12535322 38366690

[4] Eskiyurt R, Özkan B. Exploring the impact of collaborative learning on the development of critical thinking and clinical decision-making skills in nursing students: A quantitative descriptive design. Heliyon. 2024;10(17):e37198. 10.1016/j.heliyon.2024.e37198. 39295990 10.1016/j.heliyon.2024.e37198 PMC11408133

[5] Shen W, Zhu L, Lu Y. Factors influencing clinical Decision-making of nursing interns. Int J Clin Exp Med Res. 2023;7(4):579–85. 10.26855/IJCEMR.2023.10.010.

[6] Abdulmohdi N, Mcvicar A. Investigating the clinical decision-making of nursing students using high-fidelity simulation, observation and think aloud: A mixed methods research study. J Adv Nurs. 2023;79(2):811–24. 10.1111/JAN.15507;REQUESTEDJOURNAL:JOURNAL:13652648. 36412270 10.1111/jan.15507 PMC10099619

[7] Alsalamah YS, Alsalamah TS, Saad Albagawi B, Alslamah T, El Tassi A, Fawaz M. The relationship between work readiness and perceived clinical competence among graduates transitioning into professional practice. Int J Afr Nurs Sci. 2023;18:100555. 10.1016/J.IJANS.2023.100555.

[8] Beyhan A, Ergün A. Reliability and validity of the Turkish version of work readiness scale for graduate nurses among senior nursing students. J Nursology. 2022;25(4):230–7. 10.5152/JANHS.2022.222443.

[9] Dai Z, Wang J, Ma W. Adaptation and validation of the readiness for practice instrument for senior undergraduate nursing students in China. J Nurs Manag. 2023;2023(1):8345744. 10.1155/2023/8345744. 40225597 10.1155/2023/8345744 PMC11918649

[10] Jiang Z, Su Y, Meng R, Lu G, Liu J, Chen C. The effects of work readiness, organizational justice and professional identity on the work performance of new nurses: a cross-sectional survey. BMC Nurs. 2024;23(1):1–12. 10.1186/S12912-024-02420-Y/PEER-REVIEW. 39407263 10.1186/s12912-024-02420-y PMC11481288

[11] Ersoy E, Ayaz-Alkaya S. Academic self-efficacy, personal responsibility, and readiness for professional practice in nursing students: A descriptive and correlational design. Nurse Educ Today. 2024;132:106007. 10.1016/J.NEDT.2023.106007. 37922765 10.1016/j.nedt.2023.106007

[12] Ayed A. The relationship between the emotional intelligence and clinical Decision-Making among nurses in neonatal intensive care units. SAGE Open Nurs. 2025;11. 10.1177/23779608251321352. 10.1177/23779608251321352 PMC11846121 39990062

[13] Jawabreh N. The relationship between the emotional intelligence and clinical decision making among nursing students. SAGE Open Nurs. 2024;10. 10.1177/23779608241272459. 10.1177/23779608241272459 PMC11307361 39119200

[14] Btoush MR, Malak MZ, Hamaideh SH, Shuhaiber AH. The relationship between emotional intelligence, self-efficacy, and clinical decision-making among critical care nurses in Jordan. J Hum Behav Soc Environ. 2025;35(3):454–68. 10.1080/10911359.2024.2310261.

[15] Chen L, Lin Q, Xu Y, Wu L. Nursing students’ work readiness and its influencing factors before participating in a nurse residency program: a multicenter cross-sectional study. Front Med. 2024;11:1391907. 10.3389/FMED.2024.1391907/BIBTEX. 10.3389/fmed.2024.1391907 PMC11288904 39086941

[16] von Elm E, Altman DG, Egger M, Pocock SJ, Gøtzsche PC, Vandenbroucke JP. The strengthening the reporting of observational studies in epidemiology (STROBE) statement: guidelines for reporting observational studies. Lancet. 2007;370(9596):1453–7. 10.1016/S0140-6736(07)61602-X. 18064739 10.1016/S0140-6736(07)61602-X

[17] Alilu L, Namadi F, Ghodsi Astan P, Yadegar Tirandaz S. The effect of Evidence-Based education on nursing students’ clinical Decision-Making. J Res Appl Basic Med Sci. 2025;11(3):261–70. 10.61882/RABMS.11.3.261.

[18] Afra A, Bachari SS, Ban M, et al. The Design, Implementation, and evaluation of a virtual objective structured clinical examination as a tool for assessing clinical competence in nursing students. J Nurs Midwifery Sci 2025 122. 2025;12(2):e158373. 10.5812/JNMS-158373.

[19] Jenkins H. *Perceptions of Decision Making among Baccalaureate Nursing Students as Measured by the Clinical Decision Making in Nursing Scale.* 1983.

[20] Edeer AD, Sarıkaya A. Adaptation of clinical decision making in nursing scale to undergraduate students of nursing: the study of reliability and validity. Int J Psychol Educ Stud. 2015;2(3):1–9. 10.17220/IJPES.2015.03.001.

[21] Walker A, Campbell K. Work readiness of graduate nurses and the impact on job satisfaction, work engagement and intention to remain. Nurse Educ Today. 2013;33(12):1490–5. 10.1016/J.NEDT.2013.05.008. 23742716 10.1016/j.nedt.2013.05.008

[22] Xu F, Wang H, Tang S, Sun M. Factors associated with ethical decision-making ability among senior nursing interns: A National survey. Nurse Educ Today. 2025;150:106697. 10.1016/J.NEDT.2025.106697. 40120164 10.1016/j.nedt.2025.106697

[23] Maier M, Lawrie L, Powell D, Murchie P, Allan JL. Lengthy shifts and decision fatigue in Out-of-Hours primary care: A qualitative study. J Eval Clin Pract. 2025;31(2):e70050. 10. 1111/JEP.70050 40078025 10.1111/jep.70050 PMC11904385

[24] Günerigök F, Kurt FY, Küçükoğlu S. Determining nursing students’ confidence and anxiety levels in the clinical Decision-Making process: two different programme examples. J Anatolia Nurs Heal Sci. 2020;23(1):77–94. 10.17049/ATAUNIHEM.549320.

[25] Butun A, Özbay H, Ersü NF. The mediating role of self-esteem in the relationship between nursing students’ self-efficacy in paediatric medication administration and their self-confidence-anxiety levels in clinical decision-making. BMC Nurs 2025 241. 2025;24(1):734. 10.1186/S12912-025-03168-9. 10.1186/s12912-025-03168-9 PMC12211262 40598209

[26] Aboalrob W, Ayed A, Malak MZ, Aqtam I. Understanding the influence of self-concept on clinical decision-making among nurses: A cross-sectional study. PLoS ONE. 2025;20(8):e0330905. 10.1371/JOURNAL.PONE.0330905. 40853911 10.1371/journal.pone.0330905 PMC12377617

[27] Yue W, Lin Y, Ma X. From creative self-efficacy to clinical decision-making confidence: A dimensional structural equation modeling analysis through self-directed learning among nursing graduate students. Nurse Educ Today. 2025;155:106871. 10.1016/J.NEDT.2025.106871. 40957123 10.1016/j.nedt.2025.106871

[28] Bektas İ, Bektas M, Akdeniz Kudubeş A, Ayar D. Prediction of ethical decision making with professional values in senior nursing students. Perspect Psychiatr Care. 2022;58(4):2715–22. 10.1111/PPC.13112. 35575421 10.1111/ppc.13112

[29] Bisholt B, Ohlsson U, Engström AK, Johansson AS, Gustafsson M. Nursing students’ assessment of the learning environment in different clinical settings. Nurse Educ Pract. 2014;14(3):304–10. 10.1016/j.nepr.2013.11.005. 24355802 10.1016/j.nepr.2013.11.005

[30] Görücü S, Türk G, Karaçam Z. The effect of simulation-based learning on nursing students’ clinical decision-making skills: systematic review and meta-analysis. Nurse Educ Today. 2024;140. 10.1016/J.NEDT.2024.106270. 10.1016/j.nedt.2024.106270 38924975

[31] Burrell SA, Ross JG, Byrne C, Heverly MA. The effects of a Simulation-Based experience with standardized participants on learning and clinical Decision-Making related to nursing management of oncologic emergencies. J Cancer Educ. 2023;38(3):870–7. 10.1007/S13187-022-02199-Z. 35869363 10.1007/s13187-022-02199-z

[32] Akça K, Berşe S. Nursing students’ self-efficacy and clinical decision-making in the context of medication administration to children: A descriptive-correlational study. Nurse Educ Pract. 2023;72:103775. 10.1016/J.NEPR.2023.103775. 37683366 10.1016/j.nepr.2023.103775

